# Exopolysaccharide Produced by Probiotic Strain *Lactobacillus paraplantarum* BGCG11 Reduces Inflammatory Hyperalgesia in Rats

**DOI:** 10.3389/fphar.2018.00001

**Published:** 2018-01-17

**Authors:** Miroslav Dinić, Uroš Pecikoza, Jelena Djokić, Radica Stepanović-Petrović, Marina Milenković, Magdalena Stevanović, Nenad Filipović, Jelena Begović, Nataša Golić, Jovanka Lukić

**Affiliations:** ^1^Laboratory for Molecular Microbiology, Institute of Molecular Genetics and Genetic Engineering, University of Belgrade, Belgrade, Serbia; ^2^Department of Pharmacology, Faculty of Pharmacy, University of Belgrade, Belgrade, Serbia; ^3^Department of Microbiology and Immunology, Faculty of Pharmacy, University of Belgrade, Belgrade, Serbia; ^4^Institute of Technical Sciences of the Serbian Academy of Sciences and Arts, Belgrade, Serbia

**Keywords:** exopolysaccharide, *Lactobacillus paraplantarum*, probiotic, hyperalgesia, inflammation, IL-1β, iNOS

## Abstract

The aim of this study was to test the potential of high molecular weight exopolysaccharide (EPS) produced by the putative probiotic strain *Lactobacillus paraplantarum* BGCG11 (EPS CG11) to alleviate inflammatory pain in Wistar rats. The EPS CG11 was isolated from bacterial surface and was subjected to Fourier-transform infrared spectroscopy (FTIR) and thermal analysis. FTIR spectra confirmed the polysaccharide structure of isolated sample, while the thermal methods revealed good thermal properties of the polymer. The antihyperalgesic and antiedematous effects of the EPS CG11 were examined in the rat model of inflammation induced by carrageenan injection in hind paw. The results showed that the intraperitoneal administration of EPS CG11 produced a significant decrease in pain sensations (mechanical hyperalgesia) and a paw swelling in a dose-dependent manner as it was measured using Von Frey anesthesiometer and plethysmometer, respectively. These effects were followed by a decreased expression of *IL-1*β and *iNOS* mRNAs in rat’s paw tissue suggesting that the antihyperalgesic and antiedematous effects of the EPS CG11 are related to the suppression of inflammatory response. Additionally, we demonstrated that EPS CG11 exhibits immunosuppressive properties in the peritonitis model induced by carrageenan. Expression levels of pro-inflammatory mediators IL-1β, TNF-α and iNOS were decreased, together with the enhanced secretion of anti-inflammatory IL-10 and IL-6 cytokines, while neutrophil infiltration was not changed. To the best of our knowledge, this is the first study which reports an antihyperalgesic effect as the novel property of bacterial EPSs. Given the high demands of pharmaceutical industry for the replacement of commonly used analgesics due to numerous side effects, this study describes a promising natural compound for the future pharmacological testing in the area.

## Introduction

Management of pain caused either by abnormal excitation of peripheral sensory neurons or by existing local inflammation, currently leads the list of mostly demanded drugs at the pharmaceutical market. Inflammatory pain results from the increased excitation of peripheral nociceptive sensory neurons as a consequence of the accumulation of inflammatory mediators (Linley et al., 2010). Every tissue injury is followed by production of different endogenous factors released from activated nociceptors, tissue-resident sentinel cells (endothelial cells, keratinocytes, and fibroblasts) and recruited immune cells (mast cells, basophils, platelets, macrophages, and neutrophils). These factors represent a wide spectrum of signaling molecules including bradykinin, histamine, serotonin, ions (K^+^, H^+^), ATP, nitric oxide (NO), prostaglandins, pro-inflammatory cytokines (TNF-α, IL-1β, and IL-6) and growth factors (Basbaum et al., 2009; Ji et al., 2014). Some of these factors act directly on the peripheral sensory neurons affecting the specific receptors and producing a spontaneous pain, while others activate immune cells and stimulate the release of additional pain-inducing agents (Kidd and Urban, 2001). IL-1β and TNF-α represent the first cytokines formed in injured tissue and targeting these cytokines with appropriate antibodies could delay the onset of the inflammatory pain (Zhang and An, 2007). However, in spite of a major breakthrough of antibody immunotherapy, the development of novel most cost-effective drugs with potential to modulate the production of pro-inflammatory cytokines remains a significant goal in pain management.

Among the plenty of natural compounds, polysaccharides have been recognized as molecules with anti-nociceptive and anti-inflammatory activity. Bacteria from *Lactobacillus* genus possess the ability to produce different kind of polysaccharides attached to the cell surface. These polysaccharides can be associated to the bacterial cell wall forming a capsule (capsular polysaccharides) or can be loosely attached or secreted into the bacterial environment [exopolysaccharides (EPSs)] (Caggianiello et al., 2016). Lactobacilli produce both homo- and heteroexopolysaccharides exhibiting a great structural variability with different physico-chemical and biological properties (Badel et al., 2011). Beside the well-established role of EPSs in the maintenance of bacterial homeostasis and survival, the number of studies reporting the health promoting potential of EPSs has increased extensively. For instance, EPSs have been identified as the inducers of apoptosis and autophagy in the cancer cell lines (Kim et al., 2010; Di et al., 2017). Moreover, the findings of Maeda et al. (2004) revealed the effects of EPS on blood pressure and blood glucose levels, while Tang et al. (2017) demonstrated its antioxidant activity. However, one of the major pharmacological effect of EPSs lies in the modulation of immune system response and its diverse composition dictates different immunomodulatory properties (Jones et al., 2014). Given the above literature data regarding the application of EPSs in the regulation of different pathophysiological conditions, in this study, we intended to evaluate the eventual antihyperalgesic and/or antiedematous potential of the EPS isolated from *Lactobacillus paraplantarum* BGCG11 strain.

*Lactobacillus paraplantarum* BGCG11, isolated from a soft, white, artisanal cheese, produces a high-molecular-weight EPS (around 2 × 10^6^ Da) composed of glucose (75.7%), rhamnose (20.5%), galactose (2.1%), and mannose (1.7%) (Cerning et al., 1994; Zivkovic et al., 2015). Our previous studies showed that *L. paraplantarum* BGCG11 strain exhibits an anti-inflammatory effect on peripheral blood mononuclear cells (PBMC), unlike the non-EPS derivative strains which induce the higher pro-inflammatory response of PBMC. Furthermore, the upregulation of IL-10, anti-inflammatory cytokine production was detected in the culture of PBMC treated with purified EPS CG11 (Nikolic et al., 2012). Hence, it could be hypothesized that the inflammation of different etiology could be controlled by EPS CG11 treatment.

## Materials and Methods

### Bacterial Strain and Culture Condition

*Lactobacillus paraplantarum* strain BGCG11 from the laboratory collection of the Laboratory of Molecular Microbiology, Institute of Molecular Genetics and Genetic Engineering, University of Belgrade, Serbia was used in this study. The strain was grown overnight at 30°C in deMan-Rogosa-Sharpe (MRS) broth (Merck, Darmstadt, Germany).

### EPS Isolation and Purification

The EPS produced by BGCG11 strain was isolated by spreading 200 μl of overnight bacterial culture on 100 MRS plates containing 1.7% of agar (Torlak, Belgrade) in order to increase the yield of the polymer. Plates were incubated for 48 h at 30°C. After incubation time, EPS extraction was performed according to the protocol provided by Ruas-Madiedo et al. (2006), consisting in an initial extraction, ethanol precipitation and dialysis. Afterward, to reduce the content of nucleic acids and proteins, crude EPS fraction was further purified by the addition of DNAse type-I (Sigma–Aldrich, final concentration 2.5 μg/ml) for 6 h at 37°C, followed by Pronase E (Sigma–Aldrich, final concentration 50 μg/ml) treatment overnight at 37°C. Finally, the precipitation of remaining proteins was done by the addition of trichloroacetic acid (12% final concentration) at room temperature for 30 min. The mixture was centrifuged (10000 rpm, 20 min, 4°C) and the supernatant was collected and its pH adjusted to 7. The supernatant was dialyzed using 12–14 KDa molecular mass cutoff dialysis bag (Sigma–Aldrich) and finally lyophilized (Alpha 1–4 LSC Plus Freeze dryer, Martin Christ, Germany).

### Microscopy Analysis

The sample surface was observed by the OPTICA B-500MET light microscope (Optica SRL, Italy) after lyophilization and with no further preparation, by transmitted polarized and ordinary (not polarized) light. The images were collected with OPTIKAM PRO 8LT-4083.18 camera equipped with scientific-grade CCD sensor. Although not so often employed for studying biopolymer structures, the light microscopy had demonstrated to have the potential for being used as a tool in such characterization (Sanghoon and Willett, 2001).

### Fourier-Transform Infrared Spectroscopy (FTIR)

The qualitative analysis of the sample was performed by FTIR spectroscopy, which has been frequently used as a tool for detection of the structural characteristics of biopolymers (Ramesan and Surya, 2016). FTIR spectra of the samples were recorded in the range of 400–4000 cm^-1^ using a Thermo Scientific Nicolet iS10 Spectrometer equipped with Smart iTX accessory (Thermo Scientific, Inc., United States) at 4 cm^-1^ spectral resolution and 32 scans. FTIR spectra were collected in the reflection mode with the built-in diamond attenuated total reflectance (ATR) sampling technique. The OMNIC Software was used for the acquisition, processing, analyzing, and managing FTIR data in a graphical environment.

### Thermal Analysis

Thermal behavior of the sample was analyzed on two separate devices. Thermal stability and decomposition were determined using Setsys Evolution instrument (SETARAM, France). The sample was placed in alumina crucible (volume 100 μl) and subjected to the heating regime of 10°C/min from ambient temperature up to 900°C in the atmosphere of synthetic air (flow rate 50 ml/min). The corresponding differential thermogravimetric (DTG) signal and the mass loss were obtained by help of the CALISTO software taking the first derivative of the thermogravimetric analysis (TGA) signal and calculating the vertical difference on the marked horizontal points.

The potential phase transitions and the other thermally induced phenomena characteristic for polysaccharides like melting, gelatinization, swelling, or dehydration were further analyzed on the SETARAM apparatus DSC EVO 131. The accurately measured sample was placed in 30 μl sealed aluminum pans. The sample was heated/cooled from room temperature up to 200°C with a heating rate of 10°C/min and using nitrogen as a purge gas. An empty aluminum pan was used as a reference.

### Animals

Male Wistar rats (*n* = 39, weighing 200–250 g) used in the experiments were purchased from the Military Medical Academy Breeding Farm, Belgrade, Serbia. The rats were housed in the temperature-controlled room (22 ± 1°C) with food and water available *ad libitum* under a 12 h light/dark cycle. This study was carried out in accordance with the recommendations of Directive 2010/63/EU on the protection of animals used for scientific purposes, Institutional Animal Care and The Use Committee of the Faculty of Pharmacy, University of Belgrade, Serbia. The protocol was approved by the Institutional Animal Care and The Use Committee of the Faculty of Pharmacy, University of Belgrade, Serbia (No. 323-07-1193/2014-05).

### Induction of Paw Inflammation and Treatment Administration

The rats were randomly divided into four groups (*n* = 6) and inflammation of the rat’s hind paw was induced by an intraplantar (i.pl.) injection of carrageenan λ (Sigma–Aldrich; 0.1 ml/paw; 1% m/V, dispersed in 0.9% NaCl) (Morris, 2003; Stepanović-Petrović, 2012). Carrageenan-induced inflammation is followed by the development of edema and nociceptive hypersensitivity (hyperalgesia) (Morris, 2003; Vivancos et al., 2004; Stepanović-Petrović, 2012) and was used to test both the antiedematous and antihyperalgesic activity of lyophilized EPS CG11. The rats were treated intraperitoneally (i.p.), in a volume of 2 ml/kg, with 0.9% NaCl (group I, untreated control) or 3, 4, and 5 mg/kg of lyophilized EPS CG11 dissolved in 0.9% NaCl (groups II, III, and IV, respectively), 60 min before carrageenan injection.

#### Antiedematous Activity Assessment

Antiedematous activity was assessed with a plethysmometer (Ugo Basile, Comerio, Italy), by measuring the increase in paw volume following carrageenan injection, as described previously (Stepanović-Petrović, 2012). The basal paw volumes were measured before the treatment application. The post-treatment paw volumes were measured in seven time points, during 300 min after the carrageenan injection. The results are expressed as the difference (dV) between the post-treatment and the basal paw volumes according to the following formula:

dV=post-treatment volume of the inflamed paw (ml)–basal volume of the same paw (ml).

The measurements were repeated two times at each time point and the average dV of each rat was used for further calculations. The percentage of antiedematous activity (%AE) was calculated according to the following formula (Stepanović-Petrović, 2012):

%AE=[(control group average dV-dV of each rat in the test group)\(control group average dV)]×100

#### Antihyperalgesic Activity Assessment

The development of mechanical hyperalgesia following carrageenan injection and the antihyperalgesic effects of EPS CG11 were assessed by measuring paw withdrawal thresholds (PWTs) using an electronic Von Frey anesthesiometer (IITC Life Science, Woodland Hills, CA, United States) as described previously (Vivancos et al., 2004). The rats were placed in transparent boxes on the top of a metal grid and allowed to acclimatize for 30 min before testing. A plastic, semi-flexible filament coupled with a force transducer was used to deliver the mechanical stimulus. The tip of the filament was applied perpendicularly to the plantar surface of the right hind paw and the pressure was gradually increased until the rat withdrew its paw (the force, in grams, required to elicit brisk paw withdrawal was recorded on a digital screen). The average of four PWT measurements was used for further calculations.

Basal PWTs were measured before treatment application. Post-treatment PWTs were measured in seven time points, during 300 min after the induction of inflammation. The results are expressed as the difference (df) between basal and post-treatment PWTs according to the following formula (Vivancos et al., 2004):

df=basal PWT before the induction of inflammation (g)−post-treatment PWT after the induction of inflammation (g).

The percentage of antihyperalgesic activity (%AH) was calculated according to the following formula (Stepanović-Petrović, 2012):

%AH=[(control group average df-df of each rat in the test group)/(control group average df)]×100.

Immediately after the experiments animals were sacrificed using CO_2_ gas. The paws were collected, snap-frozen in liquid nitrogen and stored at -80°C until further analysis.

### Peritonitis Model

The evaluation of immunological effect of the EPS CG11 was determined in peritonitis model induced by i.p. injection of 1% carrageenan solution as previously described by Barth et al. (2016). One hour before carrageenan administration animals were treated i.p. with saline (*n* = 5, positive control) or 5 mg/kg of lyophilized EPS CG11 (*n* = 5, treatment group). Additionally, one group of animals was treated i.p. only with saline as negative control (*n* = 5). The rats were killed 4 h later and the peritoneal cavity was washed with 4 ml of RPMI medium (Gibco, Life Technologies) supplemented with 10% fetal bovine serum (FBS, Gibco, Life Technologies) to collect peritoneal cells and released cytokines. The equal volume of injected media was recovered from the peritoneal cavity in all experimental groups (*V* = 3 ml). The total cell number was determined in the Neubauer chamber in the Türk solution to exclude erythrocytes counting. The results are presented as the number of cells per ml of peritoneal fluid. The peritoneal fluid were further centrifuged (2000 rpm, 5 min) and supernatants were collected and stored on -20°C for cytokines determination, while pelleted cells were frozen on -80°C for RNA extraction.

### Enzyme-Linked Immunosorbent Assay

The levels of TNF-α, IL-6, and IL-10 were measured in the peritoneal fluid using a sandwich enzyme-linked immunosorbent assay (ELISA) according to the manufacturer’s instructions. ELISA kits for TNF-α and IL-6 were obtained from R&D Systems (Minneapolis, MN, United States) while IL-10 was from Novex Life Technologies. The results are presented as the concentrations of cytokines per ml of peritoneal fluid.

### Quantitative Real-Time PCR

The total RNA extraction from the rat paws and peritoneal cells was performed as previously described by Lukic et al. (2013). Briefly, the frozen paws were pulverized in liquid nitrogen using mortar and pestle and resuspended in denaturing solution, while peritoneal cells were directly resuspended in denaturing solution. Afterward, acid phenol (pH = 4) extraction were performed followed by isopropanol precipitation. The acid phenol extraction step was repeated two times. Reversed transcription was done using 1 μg of isolated RNA as a template (200 ng of RNA from peritoneal cells), according to the instructions of the enzyme manufacturer (Thermo Scientific). Random hexamers (Applied Biosystems) and RiboLock RNase inhibitor (Thermo Scientific) were used in the reactions. Synthesized cDNA was further amplified in 7500 real-time PCR system (Applied Biosystems) using KAPA SYBR Fast qPCR Kit (KAPA Biosystems, Wilmington, MA, United States) under the following conditions: 3 min at 95°C activation, 40 cycles of 15 s at 95°C and 60 s at 60°C. The results were normalized to endogenous controls (β-actin or GAPDH) and expressed as relative target abundance using the 2^-ΔΔCt^ method. Primers used in the study are listed in **Table 1**. All primers were purchased from Thermo Scientific.

**Table 1:** The list of primers used in this study.

Primer name	Primer sequence 5′–3′	Reference
β-Actin forward	AGCCATGTACGTAGCCATCC	Mousavi et al., 2009
β-Actin reverse	CTCTCAGCTGTGGTGGTGAA	
GAPDH forward	CCCCCAATGTATCCGTTGTG	Tanga et al., 2004
GAPDH reverse	TAGCCCAGGATGCCCTTTAGT	
TNF-α forward	AAATGGGCTCCCTCTCATCAGTTC	Peinnequin et al., 2004
TNF-α reverse	TCTGCTTGGTGGTTTGCTACGAC	
IL1-β forward	GGAAGGCAGTGTCACTCATTGTG	Tilleux et al., 2007
IL1-β reverse	GGTCCTCATCCTGGAAGCTCC	
IL-6 forward	GTGGCTAAGGACCAAGACCA	Lugrin et al., 2015
IL-6 reverse	ACCACAGTGAGGAATGTCCA	
iNOS forward	GACCAGAAACTGTCTCACCTG	Choi et al., 2012
iNOS reverse	CGAACATCGAACGTCTCACA	
CD14 forward	TCTGAGGGTCCTGGTCAACA	Lukic et al., 2017
CD14 reverse	TTGTGAGCACCGATGGACAA	


### Western Blot

Protein lysates were obtained from a paw tissue previously pulverized in liquid nitrogen using RIPA buffer and subsequently subjected to Western blot analysis as described by Dinic et al. (2017). Briefly, the extracted proteins (20 μg) were separated on 12% SDS–PAGE and transferred to 0.2 mm nitrocellulose membrane (GE Healthcare) using Bio-Rad Mini *trans*-blot system (Bio-Rad, Hercules, CA, United States). The membranes were incubated for 2 h with anti-myeloperoxidase (MPO) antibody (1:1000; Abcam) and anti-β-actin (1:1000; Thermo Scientific). The membranes were washed and incubated with appropriate HPR-conjugated secondary antibodies (goat anti-rabbit; 1:10000; Thermo Scientific and goat anti-mouse; 1:10000; Amersham Biosciences, Piscataway, NJ, United States) for 1 h at room temperature. Proteins were detected by enhanced chemiluminescence (Immobilon Western, Merck Millipore). The intensity of the bands was quantified using ImageJ software. The results were normalized to β-actin loading control.

### Statistical Analysis

The statistical analysis was performed using SigmaPlot 11 (Systat Software, Inc., Richmond, CA, United States) and SPSS 20.0 for Windows. All the results are presented as means ± standard error of the means (SEM). The differences between corresponding group means in antiedematous/antihyperalgesic activity experiment were assessed by using a two-way repeated-measures ANOVA followed by Tukey HSD test for *post hoc* comparisons (the type of treatment was the between-subject factor and time after treatment application was the within-subject factor). One-way ANOVA with the Tukey’s *post hoc* test were used to compare multiple groups in all other experiments. A *p* value less than 0.05 was considered statistically significant. Graphs were drawn in the GraphPad Prism software (trial version).

## Results

### Microscopy and FTIR Analysis of the EPS CG11

**FIGURE 1:**
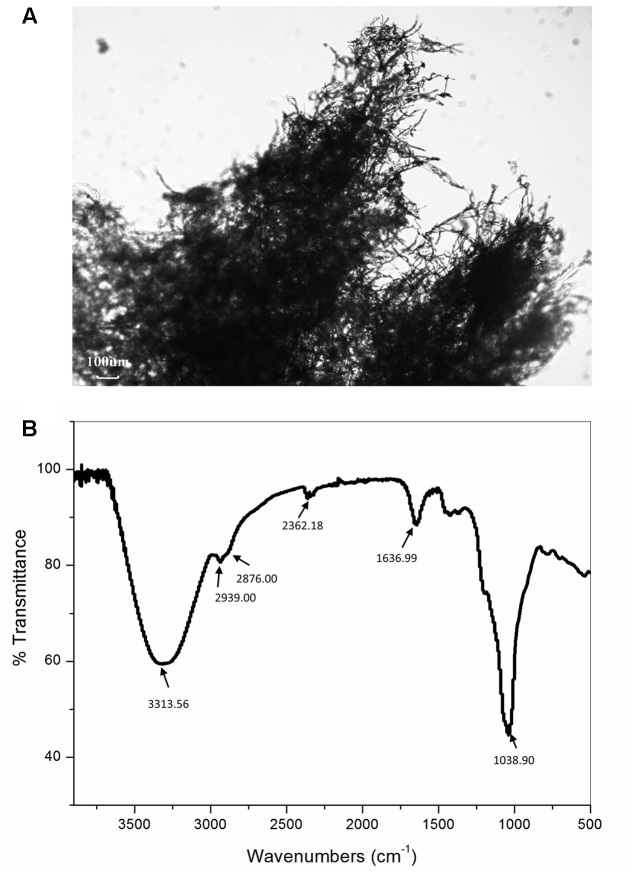
Representative light microscopy image **(A)** and Fourier-transformed infrared (FT-IR) spectrum **(B)** of the exopolysaccharide CG11 (EPS CG11) isolated from *Lactobacillus paraplantarum* BGCG11 strain.

The representative image shows that polysaccharides chains are arranged in the form of fibers with a diameter of around few microns. These fibers are further entangled with each other in a three-dimensional mesh. Such three-dimensional structure is not unusual since, among other biopolymers, the polysaccharides show the greatest chemical and structural variety (Urbani et al., 2012). This wide chemical and structural variability can be explained by the multiple hydroxyl functionality of the five- and six-carbon sugars. From the literature it is known, the replacement of one or more of such sugar hydroxyl functionalities by amine, ester, carboxylate, phosphate or sulfonate groups, leads to the frequent occurrence of tree-like branching and to the huge number of possible polymeric conformations of different solution behavior (Aspinall, 1982; Urbani et al., 2012). From the **Figure 1A** also randomly distributed spherical particles can be seen within the polymer network.

The analysis of the FTIR spectra was used to identify purified material as the polysaccharide by qualitative assessment of functional groups and chemical bonds present in the specimen. The representative FTIR spectrum is presented in **Figure 1B**. The analysis revealed the presence of carboxyl, hydroxyl, and amide groups corresponding to a typical polysaccharide. The spectrum has a broad absorption band at 3313.56 cm^-1^ assigned to –OH or –NH vibrations (Silverstein et al., 1981; Coates, 2000). The data from the literature revealed that the polysaccharides contain a significant number of hydroxyl groups, which exhibit an intense broad stretching vibration in that region (Nataraj et al., 2008). This is the characteristic absorption band of the carbohydrate ring and is responsible for the water solubility of polysaccharides. A small band at 2939 and 2876 cm^-1^ also points to C–H stretching vibrations of –CH_2_ methylene and –CH_3_ methyl groups commonly existing in hexoses, like glucose or galactose or deoxyhexose, like rhamnose. The band at 2362.18 cm^-1^ corresponds to the C–O stretch vibration. The band at 1636.99 cm^-1^ is associated with *C* = O group, while the band at 1038.90 cm^-1^ belongs to C–O stretch vibration or phosphate functional group (P-O-C stretch) and it is commonly associated with alcohols, ethers or polysaccharides (Coates, 2000). The strongest absorption band at 1038.90 cm^-1^ indicates that the substance is a polysaccharide (Nataraj et al., 2008). Also from the literature, it is known that polysaccharides generally consist of monosaccharides and some non-carbohydrate substituents (such as acetate, pyruvate, succinate, and phosphate) (Lembre et al., 2012). Apart from those peaks, in the spectra of our sample, no other peaks can be identified.

### Thermal Properties of the EPS CG11

**FIGURE 2:**
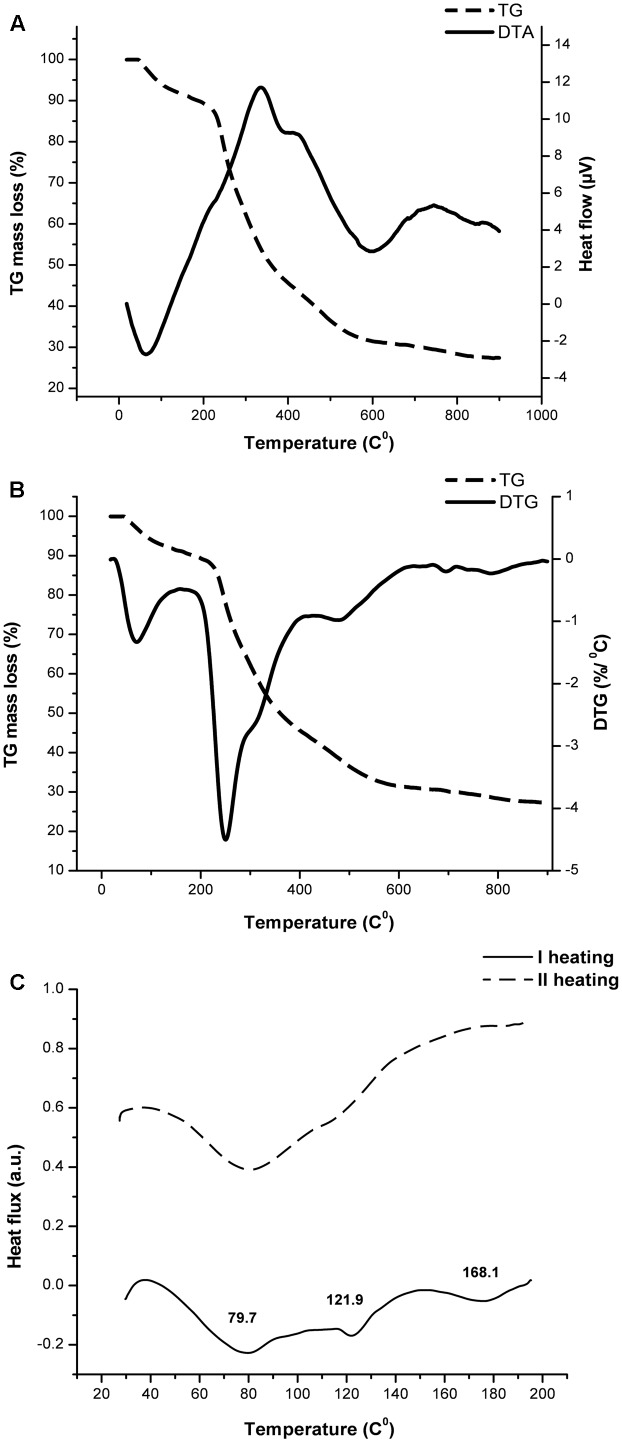
The thermal behavior assessment of exopolysaccharide CG11 (EPS CG11) isolated from *L. paraplantarum* BGCG11 strain. Diagrams represent TG/DTA **(A)**, TG/DTG **(B)**, and DSC **(C)** curves.

Substances that undergo through high temperature pharmaceutical processes must possess suitable thermal properties in order to avoid some structural deformation, degradation, or melting due to absorption or emission of the heat. Therefore, the TGA analysis was carried out to determine the thermal stability and decomposition pattern of the EPS CG11. According to Chowdhury et al. (2011), polysaccharides undergo degradation through several distinct phases. **Figures 2A,B** show TG/DTA and TG/DTG thermograms of isolated EPS CG11. At the beginning, weight loss occurs because of desorption of physically absorbed water which is then followed by the removal of structural water or dehydration. In our sample, these two processes are combined and take place in the temperature interval 46–215°C. The endothermic profile of DTA signal confirmed this theory. The calculated weight loss (Δm) for this stage is 11.5% of initial mass. The next step involves the decomposition of material which includes breaking down of C–O, C–C bonds mainly followed by the evaporation of CO, CO_2_, and H_2_O. In this decomposition process the total mass loss is 58.1% starting from 215 to 663°C with the highest rate at 250.3°C. Based on DTG signal, the decomposition can be further separated in three stages: (i) 215–264 with Δ*m* = 16.3%; (ii) 264–409°C with Δ*m* = 27.6%; and (iii) 409–663°C with Δ*m* = 14.2%. On the DTA curve, the decomposition is expressed through exothermic shoulder, which at the same time indicates the complexity of the process itself. In the remaining part of TGA curve, there is a small change in the mass of 3.1%, starting from 680 to 895°C after which the signal becomes stable. After completing the analysis, it is observed a significant amount of solid residue which could be correlated with a presence of cations (Na^+^, K^+^, and Ca^2+^) or phosphoric groups.

The differential scanning calorimetric (DSC) analysis was done in a lower temperature region, i.e., up to 200°C where the sample is stabile according to the TGA analysis. The DSC signal shown on **Figure 2C** reveals the absorption of heat at three temperatures 79.2, 121.9, and 168.1°C, respectively. The values of associated enthalpies (7–11 J/g) exclude the possibility for consideration of these events as melting processes. Also gelatinization requires a larger amount of water in the system. Most likely these endothermic events could be attributed to the removal of moisture and dehydration through evaporation, since EPS is a very hydrophilic molecule. To confirm this assumption, after cooling, the sample was subjected to the second heating regime under the same conditions. The endothermic event at higher temperature did not occur during the second run. This observation indicates that changes in the sample come only from water, without a significant influence on the polymer conformation.

### EPS CG11 Exhibits Dose-Dependent Antiedematous and Antihyperalgesic Effects in the Model of Carrageenan-Induced Paw Inflammation

**FIGURE 3:**
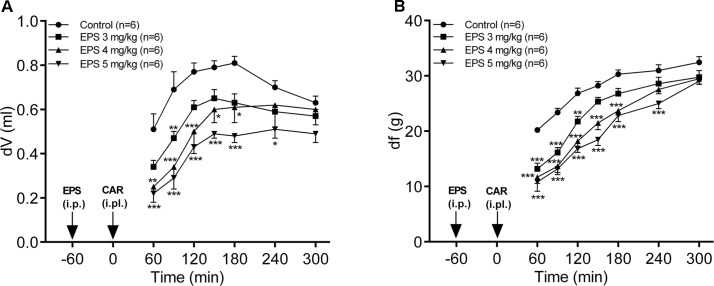
The effects of EPS CG11 (EPS) on carrageenan (CAR)-induced paw edema **(A)** and mechanical hyperalgesia **(B)**. The results are expressed as: **(A)** the difference, in milliliters, between the post-treatment and basal paw volumes (dV) and **(B)** the difference, in grams, between basal and the post-treatment paw withdrawal thresholds (df). EPS was administered i.p. 60 min before the CAR injection (denoted by arrows). Each point represents the mean ± SEM of dV (ml) or df (g). Statistical significance (^∗^*p* < 0.05, ^∗∗^*p* < 0.01, ^∗∗∗^*p* < 0.001; two-way repeated measures ANOVA followed by Tukey HSD test) was determined by comparison with the curve of the control group. i.p., intraperitoneal; i.pl., intraplantar.

The administration of EPS CG11 (3–5 mg/kg; i.p.) produced a significant and dose-dependent reduction of carrageenan-induced edema (*p* < 0.001). Maximal antiedematous effects were observed from 60 to 90 min after carrageenan treatment and were 34, 51.3, and 58% for EPS CG11 doses of 3, 4, and 5 mg/kg, respectively (**Figure 3A**).

In the case of hyperalgesia assessed by measuring PWT, EPS CG11 (3–5 mg/kg; i.p.) caused a significant decrease of carrageenan-induced mechanical hyperalgesia, in a dose-dependent manner (*p* < 0.001). The peak antihyperalgesic effects were achieved 60 min after carrageenan application and were 34.8, 42.2, and 46.5% for EPS CG11 doses of 3, 4, and 5 mg/kg, respectively (**Figure 3B**). Since the dose of 5 mg/kg produces the most promising effect, it was chosen for further analysis.

### The Expression of Inflammatory Mediators in Rat’s Paw Tissue

**FIGURE 4:**
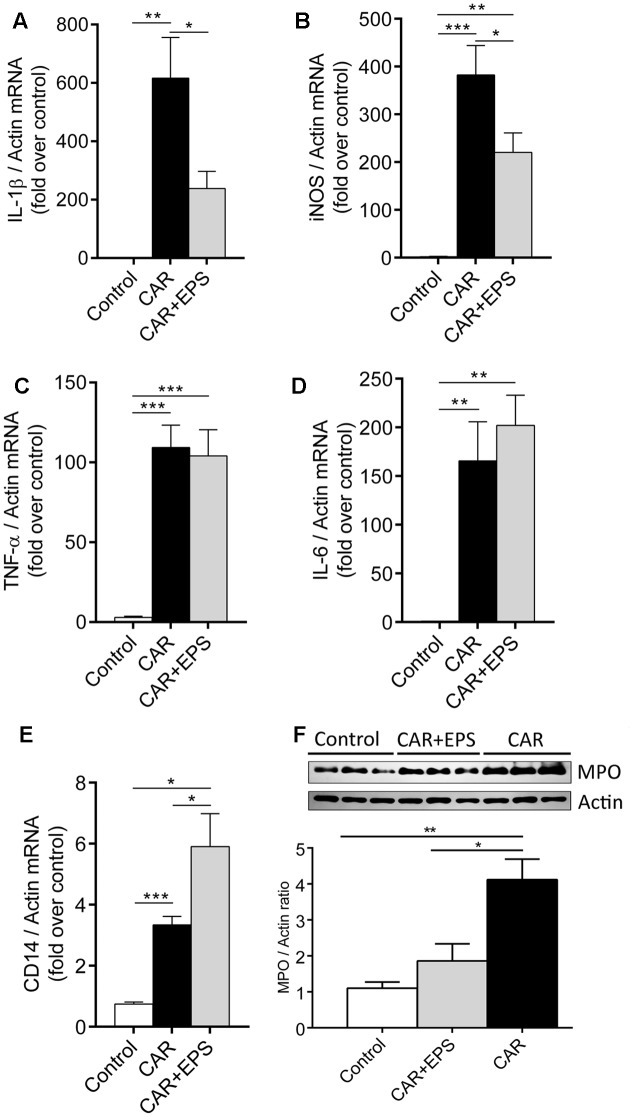
The expression of IL-1β **(A)**, iNOS **(B)**, TNF-α **(C)**, IL-6 **(D)**, and CD14 **(E)** mRNAs and representative western blot and densitometric analysis of MPO **(F)** in the paw tissue of the rats treated i.p. with 5 mg/kg of EPS CG11 (EPS) 60 min before carrageenan (CAR) injection. All values are expressed as mean ± SEM. One-way ANOVA with the Tukey’s *post hoc* test were used to compare multiple groups (^∗^*p* < 0.05, ^∗∗^*p* < 0.01, ^∗∗∗^*p* < 0.001). i.p., intraperitoneal.

Having in mind that EPS CG11 administration alleviated the carrageenan-induced rat paw edema, we proceeded with the analysis of gene expression of pro-inflammatory cytokines (TNF-α, IL-1β, and IL-6) as well as iNOS to determine whether the transcription of some of these genes could be potentially altered by the EPS CG11. The effect of the EPS CG11 on gene expressions in the rats paw tissue is presented in **Figure 4**. As expected, the carrageenan injection in the rats paw tissue significantly elevates mRNA levels of all tested genes compared to saline administration. The mRNA expression of *IL-1*β and *iNOS* genes in the paw tissues of rats treated with 5 mg/kg of EPS CG11/carrageenan were significantly lower compared to the expression levels of these genes in the rats injected only with carrageenan (*p* < 0.05). More precisely, *IL-1*β expression showed 2.6 fold reduction when EPS CG11 was administered, while *iNOS* mRNAs were reduced 1.7 times (**Figures 4A,B**). However, EPS CG11 administration did not change the transcription levels of *TNF*-α and *IL-6* genes which retained a high mRNA levels induced by carrageenan (**Figures 4C,D**). These results indicate the potential of i.p. injected EPS CG11 to suppress inflammatory response via regulation of IL-1β production on periphery.

Further, we assessed the expression of MPO, an enzyme used as a marker of neutrophil recruitment and activation, and CD14, a macrophages tissue marker. The results revealed the reduced level of MPO enzyme and the elevated level of *CD14* mRNA in the paw tissue of animals co-treated with EPS CG11/carrageenan compared to carrageenan controls, suggesting that i.p. injected EPS CG11 suppress the function of neutrophils in the paw tissue, but also pointed out the potential contribution of macrophages to the suppression of inflammation (**Figures 4E,F**).

### EPS CG11 Shows the Immunosuppressive Properties in the Peritonitis Model

**FIGURE 5:**
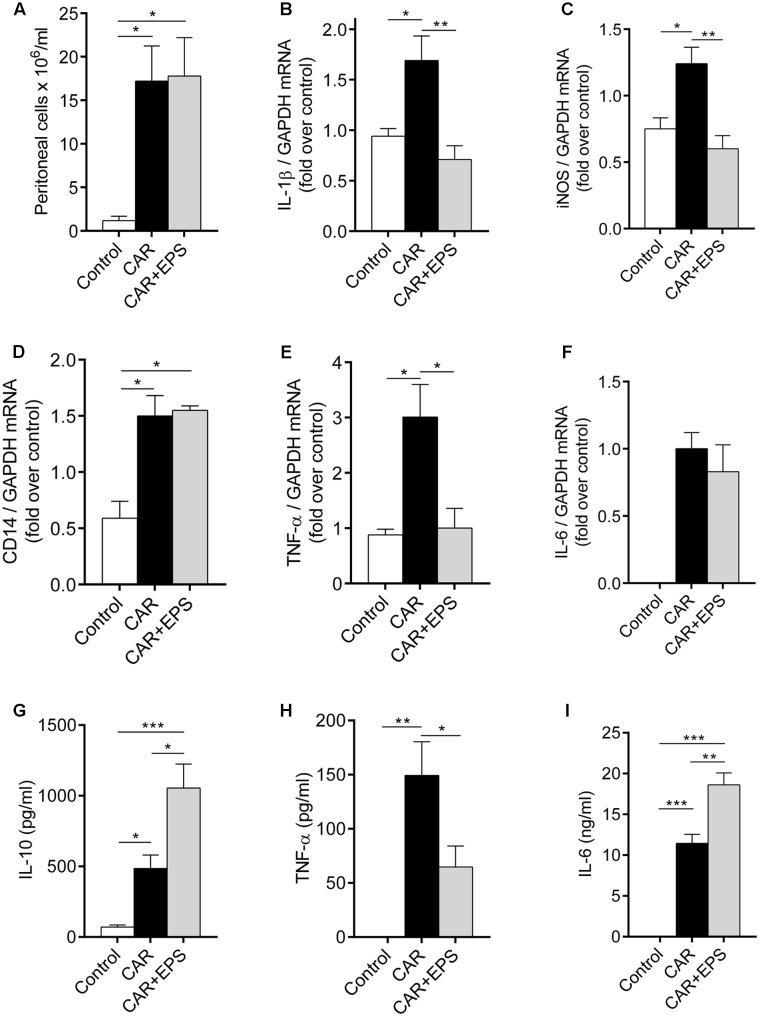
Assessment of immunosuppressive properties of EPS CG11 in the peritonitis model. The number of cells infiltrated in the peritoneal cavity **(A)**, expression of IL-1β **(B)**, iNOS **(C)**, CD14 **(D)**, TNF-α **(E)**, and IL-6 **(F)** mRNAs in the peritoneal cells and the levels of IL-10 **(G)**, TNF-α **(H)**, and IL-6 **(I)** cytokines in the peritoneal fluid after i.p. administration of 5 mg/kg of CG11 EPS (EPS) 60 min before i.p. carrageenan (CAR) injection. The results are presented as means ± SEM. For the comparison of multiple groups, one-way ANOVA with the Tukey’s *post hoc* test were used (^∗^*p* < 0.05, ^∗∗^*p* < 0.01, ^∗∗∗^*p* < 0.001). i.p., intraperitoneal.

To confirm our hypothesis on the regulation of cytokines production by EPS CG11, a peritonitis model was used. As the previous studies have reported, carrageenan caused the migration of immune cells, predominantly neutrophils, into the peritoneal cavity and the total cell number was significantly higher in the animals receiving carrageenan relative to healthy control animals (*p* < 0.05). However, the i.p. injection of EPS CG11 treatment (5 mg/kg) did not affect carrageenan induced-immune cells migration (**Figure 5A**). Additionally, in the contrast to the paw tissue, i.p. injected EPS CG11 did not affect *CD14* mRNA level in the infiltrated cells induced by the carrageenan administration (**Figure 5D**).

We further used the qPCR analysis to investigate the changes in the expression of *IL-1*β, *TNF*-α, *IL-6*, and *iNOS* genes in the infiltrated cells. Interestingly, the mRNA levels of *IL-1*β, *TNF*-α, and *iNOS* in EPS CG11/carrageenan treated animals were significantly lower compared to only carrageenan treated animals (*p* < 0.01; *p* < 0.05; *p* < 0.01, respectively), while the transcription of *IL-6* gene was on the same level between these two groups of animals (**Figures 5B,C,E,F**). In other words, the levels of *IL-1*β, *TNF*-α and *iNOS* mRNAs was reduced for 2.4; 3 and 2 times, respectively, compared to carrageenan controls.

The results of the ELISA measurements revealed that the EPS CG11 administration led to the significant reduction of high level of TNF-α induced by carrageenan (*p* < 0.05) (**Figure 5H**). Interestingly, the higher levels of IL-10 and IL-6 in peritoneal fluid in the animals treated both with EPS CG11 and carrageenan compared to the animals received only carrageenan or healthy control animals were detected (**Figures 5G,I**). Finally, carrageenan itself has led to significantly higher levels of all three cytokines, while in the healthy control animals the levels of IL-6 and TNF-α were under the detection limit (**Figures 5G–I**).

Therefore, all the above mentioned results led us to conclusion that immunosuppressive mediators were predominantly present in the peritoneal cavity when EPS CG11 was administered.

## Discussion

A huge diversity of microbial world enables us to constantly search new molecules with a potential to interact with the host cells. Presently, between 20 and 50% of medications originate from natural sources including bacteria (Kingston, 2011). Besides antimicrobial molecules usually found in bacteria, cell surface macromolecules as well as bacterial metabolites have been extensively studied due to their key role in the interaction with host receptors (Dinic et al., 2017). Different modifications (e.g., glycosylation) of surface molecules and/or the presence of strain exclusive molecules (e.g., EPSs) dictate strain specific effects, which explain applications of numerous bacterial strains as probiotics (Lebeer et al., 2010; Zivkovic et al., 2016). It’s well-documented that EPS production is involved in the probiotic beneficial effects. Therefore, in the light of rising application of bacterial EPSs, here we demonstrate the novel ability of EPS CG11 to suppress a pain sensation via regulation of inflammatory response.

Previous studies reported that strain *L. paraplantarum* BGCG11 produces ropy, big-size polymer around 2 × 10^6^ Da, mainly composed of glucose and rhamnose with the traces of galactose and mannose, but further information regarding stability of the polymer is missing (Cerning et al., 1994; Zivkovic et al., 2015). As one of the important steps in drug design and development is the investigation of thermal behavior of new molecules in order to collect stability information for adequate pharmaceutical processing (Shamsipur et al., 2013). According to the literature data, the thermal stability of EPSs from various bacterial sources may vary. The TGA curve of EPS CG11 showed that maximal degradation temperature of the polymer was 250.3°C, while the overall mass loss at the end of the analysis was 72.7%. This is in accordance with the decomposition temperature reported for EPSs isolated from *L. plantarum* and *L. kefiranofaciens* (Wang et al., 2010; Ahmed et al., 2013). On the other hand, low enthalpy values (7–11 j/g) of EPS CG11 measured in DSC analysis, confirmed that this EPS molecule did not undergo phase transition processes unlike KF5 EPS produced by *L. plantarum* which melting temperature was 86.35°C, with the enthalpy value of 133.5 J/g (Ahmed et al., 2013). These different observations in the thermal behavior of these two EPSs could be explained by different structures and monosaccharide composition of the polymers. Hence, it can be said that EPS CG11 possesses good thermal properties and could undergo through various technological processing where temperature reach 150°C (Ahmed et al., 2013).

It has been shown that the crude polysaccharides as well as sulfated ones isolated from lichens and seaweeds exhibit potential to alleviate inflammatory pain in rodents by suppressing IL-1β and/or TNF-α production (Chaves et al., 2013; Córdova et al., 2013). The results obtained in our study are in accordance with the literature data regarding the polysaccharide application as pain relief agents. The dose-dependent reduction of pain sensations as well as paw edema was noticed after the administration of EPS CG11 in carrageenan-induced paw inflammation in rats. The carrageenan administration evokes biphasic edema. The first phase of edema last for 1 h and is reflected in the release of histamine, bradykinin, serotonin and cyclooxygenase products (8-iso-prostaglandin F_2α_; 8-iso-PGF_2α_ and prostaglandin E_2_; PGE_2_). During the second phase, which last for the next 2 h, neutrophil infiltration, the liberation of cytokines and NO occur, while prostaglandins production continue (Vinegar et al., 1969; Ozdol and Melli, 2004). Taking into account that the effect of EPS CG11 was the strongest for 2 h after the carrageenan administration we could assume that EPS CG11 influences both phases of inflammation.

Based on these findings, we analyzed the influence of EPS CG11 on a release of inflammatory mediators important for hyperalgesia and inflammation. Numerous studies focused on the pro-inflammatory cytokines showed that injections of TNF-α, IL-1β, and IL-6 produce both mechanical and thermal hyperalgesia. On the other hand, a release of potent anti-inflammatory cytokine IL-10 attenuates spinally mediated pain, counter-regulating the synthesis of pro-inflammatory cytokines (Zhang and An, 2007). In the paw tissue of EPS CG11/carrageenan treated rats, EPS CG11 caused a downregulation of *IL-1*β and *iNOS* mRNA levels, while the levels of *TNF*-α and *IL-6* mRNAs were not changed compared to carrageenan controls. The same effect was observed by Córdova et al. (2013) reporting that the analgesic effect of glucomannan depended on the inhibition of IL-1β release, without the affection of TNF-α production in the sciatic nerve. Additionally, as the transcription of *iNOS* gene is regulated by IL-1β (Pautz et al., 2010), the decrease in *iNOS* mRNA levels measured in the paws of animals treated with EPS CG11/carrageenan compared to carrageenan controls is probably the consequence of *IL-1*β downregulation. Taking into account that the expression of cyclooxygenase-2 (COX-2), which is responsible for the synthesis of prostaglandins (8-iso-PGF_2α_ and PGE_2_) during the first phase of carrageenan inflammation, is stimulated by IL-1β production, we could assume that the decreased level of *IL-1*β mRNA caused by EPS CG11 contributed to its anti-inflammatory effect by acting on the prostaglandins synthesis in the beginning of the inflammatory process (Molina-Holgado et al., 2000). Also, the decreased MPO level detected in the paw tissue in the group of animals receiving EPS CG11/carrageenan suggested that anti-inflammatory effect of EPS CG11 could be the consequence of impaired neutrophils’ function in the paw.

In order to understand the role of EPS CG11 in the modulation of cytokines’ production we additionally used the carrageenan-induced peritonitis model suitable for the investigation of leukocyte migration (Barth et al., 2016). As was noticed in the paw tissue, the expression of *IL-1*β and *iNOS* mRNAs showed approximately the same level of reduction in the peritoneal cells after EPS CG11/carrageenan administration compared to carrageenan controls. Additionally, level of TNF-α, as well as the transcription of TNF-α gene, was significantly reduced in the same group of animals, together with the increased level of anti-inflammatory cytokine IL-10. In the case of IL-6, which is categorized as either anti-inflammatory or pro-inflammatory cytokine, under various circumstances (Zhang and An, 2007), there is a discrepancy between its mRNA and protein level in peritoneum of EPS CG11/carrageenan treated rats. Although, the protein level of IL-6 was elevated in the peritoneum, the mRNA level was unchanged and in accordance with that obtained in the paw tissue. Given that, the cell infiltration in the peritoneal cavity was the same irrespectively of EPS CG11 treatment, it could be concluded that the reduction of pro-inflammatory cytokines was not related to lower infiltration of neutrophils. A similar effect was exhibited by cyanidin-3-glucoside, an anthocyanin, when administered prophylactically, as we did with EPS CG11, in the carrageenan-induced peritonitis model (Hassimotto et al., 2013). More precisely, the authors noted that cyanidin-3-glucoside given 30 min before carrageenan alleviated inflammation by suppressing COX-2 expression and PGE_2_ synthesis, with no effect on neutrophil influx.

Further, the elevated level of *CD14* mRNA in the rat hind paw, pointed to the possibility that EPS CG11 exhibits its anti-inflammatory effect by acting on macrophages. Therefore, based on the higher secretion of IL-10 and the lower levels of *IL-1*β and *iNOS* mRNAs observed in EPS CG11/carrageenan treated rats in the peritonitis model, it could be assumed that the intraperitoneal injection of EPS CG11 probably influenced the peritoneal macrophages to acquire the immunosuppressive properties. Together with the report of Bhaumik et al. (2001) that macrophages migrate from the peritoneal cavity to the subcutaneous tissue, above results suggest the potential involvement of peritoneal macrophages in the resolution of paw inflammation. However, as neutrophils also express CD14 molecule, this hypothesis about macrophages infiltration/migration needs to be confirmed in further experiments. Altogether, the differences in cytokine production and the cells migration between the paw tissue and the peritoneal cavity are most probably due to the differences of EPS CG11 and carrageenan application route in these two models of inflammation. So, in the inflammatory pain model, only cells that are exposed to the EPS CG11 are those migrating from the peritoneum, while all other cells in the inflamed paw are exposed only to carrageenan. At contrary, in model of peritonitis, resident as well as all infiltrated cells are exposed to the EPS CG11 and carrageenan simultaneously.

Finally, considering the results obtained in this study, we can conclude that EPS CG11 is highly promising antihyperalgesic molecule with good physico-chemical properties which makes it a potential candidate for further investigation and drug-development.

## Author Contributions

MD: performed the main work, conception and design of the experiments, analyzed and interpreted the data, and drafted the work; UP: performed *in vivo* experiments, analyzed and interpreted the data, and made a part of the draft related to antihyperalgesic and antiedematous experiments; JD and MM: conception and design related to immunology, supervised the work, analyzed and interpreted the data, and critically revised the manuscript; RS-P: conception and design related to antihyperalgesic and antiedematous experiments, supervised the work, analyzed and interpreted the data, and critically revised the manuscript; MS and NF: performed part of the work related to physico-chemical analysis, analyzed and interpreted the data, and made a part of the draft; JB, NG, and JL: supervised the work, analyzed and interpreted the data, and critically revised the manuscript. All authors finally approved the version to be published and agreed to be accountable for all aspects of the work in ensuring that questions related to the accuracy or integrity of any part of the work are appropriately investigated and resolved.

## Conflict of Interest Statement

The authors declare that the research was conducted in the absence of any commercial or financial relationships that could be construed as a potential conflict of interest.
